# Therapeutical Action of Arsenic

**Published:** 1872-02

**Authors:** J. S. Cassidy


### THERAPEUTICAL ACTION OF ARSENIC.

From the Minutes of the Covington and Newport Medical Society, January 9th, 1872.

The reading of the regular paper being in order, Dr. Cundell Juler read a paper entitled "An Inquiry into the Nature of the Therapeutical Action of Arsenious Acid." After briefly reviewing and combatting the generally recognized therapeutical actions of arsenic, he drew the attention of the Society to what he termed the *true* therapeutical action of the drug, which he held was that of a nerve sedative. He pointed out the influence which arsenic exercised upon the nervous centers, in regulating their forces, controlling those spasmodic conditions of the vaso-motor nerves, that tended to an irregular supply of blood to different parts of the body, and led to the well known results of that condition. Its action, therefore, as a nerve sedative, although not hitherto considered in that light, was highly appreciated in the treatment of diseases of the skin—affections which are in a great measure occasioned, and, like eczema, may be artificially produced by irritation of the peripheral nerve fibres, giving rise to reflex morbid results upon the parts irritated, or upon distant parts, which are sympathetic one with the other.

To secure its sedative action upon the nerves, it was also given in epilepsy and cholera, as well as in many convulsive and spasmodic affections, where, by reason of the irregular supply of blood, the nervous system eventually becomes functionally deranged. Also as an antiperiodic in agues, where, in consequence of a peculiar effect, long continued upon the cuticular nerve filaments, a neurosis is initiated, which, by repeating itself in the form of chills, &c., becomes established, like epilepsy, asthma, and other neuroses—a disease often needing, besides arsenic, the exercise of some moral influence or diversion to complete the cure, and, if neglected, giving rise to diseases of a far graver character. As a sedative, he also advocated its use in instances where morbid foci of disturbed nervous power have become developed, with stasis in the capillary network, accompanied by morbid changes in the tissues themselves, independent of nerve or blood influence, but causing an irritation of surrounding nerve filaments, producing increase of temperature, sometimes fever and disease of the trophic nerve centers, as in the case of furunculus, carbuncle, malignant pustule, &c. Arsenious acid, being exercised upon the nerve tubes, assists, by its sedative influence, in restoring to the ganglionic nerve centers their lost power; assuaging the irritative fever, by deadening the sensory nerve filaments, restoring the digestive function, and changing to health the morbid condition of the capillaries, as well as the affected tissues. Again, by acting as a sedative to the nerves supplying depurative organs, arsenious acid relieves the small blood vessels of their undue muscular contraction, favors the necessary changes in the blood and its corpuscle, gives rise to increased action in the glands or organs, and thus increasing in quantity the discharges from the uterus, kidneys, salivary glands, &c. Arsenic is also curative in ulcer of stomach, irritable dyspepsia, morning lassitude, with loss of appetite, in many diseases of the membrane of the bowels, in dysentery, hemorrhoids, and by some physicians deemed of great value in cholera, in consequence, as the author maintained, of the sedative influence which the medicine has in subduing the irritation of the trophic and vaso-motor nerves, checking the abnormal nutrition which has been preventative of cure, and by allaying any exaggerated sensibility of the sensory nerve fibres. Acting clearly as a nerve sedative, it restored painful peripheral nerve fibres—as in neuralgias—to health and comfort. But if the administration of arsenic were continued beyond the period of the exercise of its sedative effect, paresis or paralysis of nerve power would ensue, and then a complete change of phenomena to those exhibited while its sedative action upon the nerves is manifested would follow. These toxical effects upon the system were shown to be similar to those produced by digitalis, a well known vegetable nerve sedative if given in sufficient doses to develop its poisonous effects.

In the second portion of his paper, Dr. Juler drew attention to what he conceived was a clue to the right administration of the drug. The very derangements and morbid conditions of the organism developed by the medicine, when given continuously, in sufficient doses, were similar in character with those derangements and morbid conditions of the organism in which the administration of the medicine has proved most beneficial. To sustain him in this view of the action of the drug, he quoted from the writings of many authors. He concluded his discourse by asking the members of the Society to disabuse their minds of all prejudice engendered by book knowledge; to forget all that its enemies had unjustly written against the use of the medicine; to cooperate with him in this important and interesting inquiry; to watch the effect of arsenic upon the system, when given by them, as they would that of some newly discovered herb, whose medicinal action was to be duly noted and recorded, and to remember that arsenic, no more than digitalis, is an accumulative poison; that certain effects may be produced upon the nervous system more or less obvious, but that saturation of the system with arsenic is an improper term. The smallest fatal dose in an adult placed on record, is stated to have been thirty grains of the powdered white arsenic, equal to nearly eight ounces of Fowler's solution. The man expired in six days; and cases have been known to recover after the injection of half an ounce of arsenious acid. It is so rapidly extruded from the system when taken medicinally, that arsenic has been shown by Mr. Hunt, of the Western Dispensary for Diseases of the Skin, in London, to be eight or ten times safer than almost any other medicine.

In the practice of my profession as a dentist, I acknowledge to have always been at a loss to account for the peculiar effect of arsenious acid, when employed locally, for the purpose of devitalizing the dental pulp, its direct application causing necrosis of the nerve in from six to forty-eight hours, without any evidence whatever of escharotic influence.

Dr. Juler's assertion, that arsenic is a nervine sedative, seems to me to be the correct explanation of its potency in dental operations, its place among the tonics and alteratives being wholly incompatible with the results in question.

J. S. Cassidy, M. D., D. D. S., *Secretary.*

